# How do long COVID patients perceive their current life situation and occupational perspective? Results of a qualitative interview study in Germany

**DOI:** 10.3389/fpubh.2023.1155193

**Published:** 2023-03-09

**Authors:** Tim Schmachtenberg, Frank Müller, Jennifer Kranz, Anita Dragaqina, Greta Wegener, Gloria Königs, Sascha Roder

**Affiliations:** ^1^Department of General Practice, University Medical Center Göttingen, Göttingen, Germany; ^2^Department of Rheumatology and Immunology, Hannover Medical School, Hannover, Germany

**Keywords:** long COVID, life situation, occupational perspective, social aspects, work, qualitative research, interview study, quality of life

## Abstract

**Introduction:**

Many people experience persistent or new-onset symptoms such as fatigue or cognitive problems after an acute infection with COVID-19. This phenomenon, known as long COVID, impacts physical and mental wellbeing, and may affect perceived quality of life and occupational perspectives likewise. The aim of this study is to gain a deeper understanding of how people with long COVID experience health-related restrictions in their daily life and their occupational situation, and to identify key challenges they face.

**Methods:**

Guided qualitative interviews were conducted with 25 people with long COVID. The interviews were transcribed according to Dresing/Pehl and Kuckartz and analyzed using qualitative content analysis. Afterward, a systematic comparison of the data and a reflection under consideration of lifeworld-theoretical approaches (Berger and Luckmann) were carried out.

**Results:**

The interviews revealed that many participants have severe symptoms which strongly impair them in perform daily and work-related activities, and in their personal interests. Many interviewees already reach their stress limit during routine household activities or childcare. Of the 25 participants, 19 experienced limitations in pursuing leisure activities, and 10 of the 23 interviewees with jobs reported being on sick leave for several months. Several respondents who had vocational reintegration are still affected by ongoing symptoms that affect their work performance considerably. This leads to uncertainty, role conflicts, a decline in social contacts, and decreased incomes, which contribute to an impairment in their quality of life.

**Conclusions:**

This study shows the huge need for specific support for people with long COVID in different areas of life. To prevent people with long COVID from finding themselves in social and economic precarity, decision-makers should develop strategies to systematically support them in their sustainable reintegration into the workforce. The focus should be on creating long COVID-sensitive workplaces, compensating for decreased incomes, and improving access to relief services such as vocational reintegration. We argue, that a shift of perspectives is necessary and that long COVID should be considered rather as a “social disease” with considerably impairments in the social life of those affected.

**Trial registration:**

The study is registered in the German register for clinical trials (DRKS00026007).

## Introduction

More than 750 million people worldwide have been infected with SARS-CoV-2 (severe acute respiratory syndrome coronavirus type 2) (1). In Germany, public health authorities reported ~38 million infection cases since the beginning of the pandemic (as of 30 January 2023) (2). A recent systematic review suggests that persistent and long-lasting symptoms following acute COVID-19 disease occurs in at least 7.5% of non-hospitalized adults and considerably higher rates have been shown among hospitalized patients (3). While terms like “post-acute COVID-19,” “long haulers,” “post-COVID-19,” and others have been introduced, we use the term “long COVID” in the following as proposed by the German Robert Koch Institute (RKI) (4). In accordance with the UK National Institute for Health and Care Excellence (NICE) (5), the RKI defines long COVID as health symptoms that persist beyond the acute phase of illness of an SARS-CoV-2 infection of 4 weeks or are new onset (4). Long COVID is considered a multisystem disease with a heterogeneous clinical picture (6): Common symptoms of long COVID are fatigue, cognitive problems (especially difficulties in concentrating, often referred to as “brain fog”), shortness of breath, headache, joint and muscle pain, and emotional distress (e.g., symptoms of anxiety or depression) (6–11). While long COVID generally may affect anyone infected with COVID-19, female gender, and hospitalization during acute infection, are associated with an increased risk (7, 12–14). Immunological processes that cause inflammation have been proposed as a likely mechanism of long COVID (12). Currently, there is no known effective and approved cure (7, 15). Previous research has highlighted reduced quality of life, prolonged absence at work, and other unfavorable impacts on working life among persons with long COVID (9, 16, 17). This research applies a qualitative approach to gain a deeper understanding of how people with long COVID perceive the impact of their physical and cognitive symptoms on key aspects of their daily lives such as domestic activities, personal interests, and their occupational situation. The study supplements the existing literature with a description of “social symptoms” of long COVID and the impact of the disease on social functioning, perception of own identity, and roles in the context of partnership, family, and work environment of affected individuals.

## Methods

This study is part of the research project “DEFEnse Against COVID-19 STudy—Looking forward” (DEFEAT Corona) (18), a multicenter project that aims to improve the understanding of the long COVID syndrome and the long-term effects of the pandemic. Within this project, we conducted a qualitative interview study using an interview guide that was iteratively developed. Topics of the interviews included health status, cognitive and physical impairments, experiences with health care provision, daily-life experiences, social situation, personal interests, experiences at work, and life perspectives. For this work, we analyzed selected aspects of these complex interviews. These aspects included the living situation, daily routine, occupational situation, and leisure activities. This publication follows the consolidated criteria for reporting qualitative research (COREQ) (19). The study received approval by the research ethics boards of Hannover Medical School (No. 9948_BO_K_2021) and University Medical Center Göttingen (39/8/21) and written informed consent was obtained from all participants.

### Inclusion and exclusion criteria

Inclusion criteria for this study were having long COVID as defined by RKI/NICE (i.e., symptoms that develop during or after an infection consistent with COVID-19, continue for more than 4 weeks and are not explained by an alternative diagnosis), aged 18 or older, and consent to participate in either video teleconferencing or face-to-face interviews. Individuals who did not have persistent COVID-19 symptoms or whose SARS-CoV-2 infection had occurred < 4 weeks ago, under aged persons, people who did not consent to participate in a video teleconference or face-to-face interview, and people whose cognitive or physical impairment was too severe for them to attempt an at least 1-h interview had to be excluded for practical and ethical reasons.

### Recruitment

Recruitment strategy included (a) sending invitation letters (*n* = 130) to people who already had participated in a previous subproject (20) of DEFEAT-Corona, (b) handing out leaflets with study information at general practices in Lower Saxony and the Office of the Public Health Authority in Göttingen, and (c) posting on the project homepage and the social network channels (Instagram and Facebook) of the University Medical Center Göttingen. Additionally, (d) participants suggested other people to participate (snowball sampling). The invitation letters and flyers explicitly invited people with long COVID symptoms to participate. Recruitment efforts occurred in January and February 2022. Interested individuals were contacted *via* phone or e-mail prior to the actual interview to verify eligibility criteria and provide general information about the study. The participants were informed of the study's objectives and the professional background of the interviewer. Through email correspondence and on documents prepared by the researchers, recruited persons gave written informed consent to participate in the guided interviews and to publish the data. Participants were asked to print out and send back a signed consent form prior to the interview either as a scanned version by email or mail. In terms of sample size, we followed existing literature on good qualitative research practice, which recommends conducting at least 12 interviews (21–23). Participants received compensation for participating in the interviews of 40 EUR (~40 USD).

### Development of interview guideline

On October 29th 2021, a workshop with six researchers was conducted at the Department of General Practice to specify research questions and subsequently developed an initial version of an interview guideline. As leading research questions were identified: (1) How does long COVID symptoms impact daily responsibilities and activities? (2) How does long COVID affect personal interests and leisure activities? (3) How do long COVID symptoms impact work activities? (4) How do people with long COVID experience support and impairments at their workplace? This general set of research questions were refined into sub-questions and comprised to an interview guideline. Additionally, a closed-question section was added to the guideline to collect sociodemographic information about the interviewees. This first version of the guideline was then used in four pretest interviews after which the guideline was further refined according to additional topics that were raised by the interviewees and considered as important. The changes concerned areas of the interview that are not reported in this work. The final interview guideline comprised 26 open-ended questions. This final version of the guideline is presented in the Supplementary material. To gain authentic insights into the participants' current life situation, the researchers decided not to provide them with the interview guideline in advance.

### Data collection

The interviews were carried out between January and May 2022. With a few exceptions, the interviews were conducted using video teleconferencing software Zoom (Zoom Video Communications, Inc., San Jose, CA, USA). By using video teleconferencing we were able to include people with limited mobility who otherwise would not have participated. The participants connected to these online meetings from their homes. Additionally, four interviews were conducted in a seminar room at the Department of General Practice in Göttingen. These interviews were carried out during a period with lower infection rates in April/May 2022 and were conducted to include interview aspects that may be limited to cover in video teleconferences. These are, for example, the understanding of the situation the interviewees are currently in and the visibility of non-verbal communication beneficial for the interaction (24). The interviews were carried out with interviewer and participant present. In some interviews, a graphic artist joined the videoconference interview as part of an experimental approach of an graphical interpretation of study results (25). All interviews were conducted by an experienced male sociologist (SR). The teleconferencing interviews were digitally audiorecorded, and for the face-to-face interviews a digital recording device was used. Additionally, the interviewer noted peculiarities and thoughts that emerged during the interviews.

### Data evaluation

The interview recordings were transcribed in terms of content-semantics following Dresing/Pehl and Kuckartz (26, 27) and all transcripts were reviewed for accuracy. Subsequently, the interviews were analyzed using qualitative content analysis (28, 29). A team of two medical sociologists (TS and SR) who are experienced in qualitative research, and three study assistants (JK, AD, and GK), developed a category system in an iterative and discursive process. A combination of deductive and inductive categorization was used in the design of the category system. In a first step main catagories were derived deductively from the interview guideline. Additionally, emerging topics were identified in an open coding process. Hereafter, subtopics were developed using axial coding. The content analysis process was repeatedly discussed within the study team and a codebook was developed including definitions of the codes, exemplar quotes, paraphrases, and semantic meaning. Subsequently, coding and summaries in the form of paraphrases and generalizations were performed. Table 1 provides an overview of all categories generated in this study. The category system consists of five main categories, 18 top categories, and 15 subcategories. This work focuses on the main category personal history, four of its top categories, and five subcategories (highlighted in bold in the table). In a final analysis step, generalizations from all interviews were summarized in a table and systematically compared and interpreted. The MAXQDA software version 20.0.8 (VERBI Software GmbH, Berlin, Germany) was used for coding and analyzing the interviews.

**Table 1:** Category system.

**Main categories**	**Top categories**	**Subcategories**
1. COVID-19 Pandemic		
	1.1. Personal view on the COVID-19 pandemic	
	1.2. Attitude toward COVID-19 regulations and handling of COVID-19 measures	
		1.2.1. Self-reflection of behavior during COVID-19 pandemic
	1.3. Public communication	
	1.4. Criticism of society	
2. Social environment		
	2.1. Persons in need of care	
	2.2. Support and consideration	
	2.3. Partnership	
		2.3.1. Stresses in the partnership
		2.3.2. Relief in the context of a partnership
3. Personal history		
	3.1. **Living situation**	
		3.1.1. **Changes through COVID-19 measures**
	3.2. **Daily routine**	
	3.3. **Occupational situation**	
		3.3.1. **Field of activity**
		3.3.2. **Home office**
		3.3.3. **Support from the work environment**
		3.3.4. **Work satisfaction**
	3.4. **Personal interests**	
	3.5. Vacations	
	3.6. Church affiliation	
4. Health situation		
	4.1. Current state	
	4.2. COVID-19 infection	
		4.2.1. Assessment of personal risk
		4.2.2. Consequences of COVID-19 infection
		4.2.3. Improvement of the complaints
		4.2.4. Therapeutic measures
	4.3. COVID-19 vaccination	
		4.3.1. Immunization education
		4.3.2. Side effects of COVID-19 vaccination
	4.4. Healthcare orientation	
	4.5. Health behavior	
		4.5.1. Strategy development regarding health restrictions
5. Questions for the interviewer		

Categories analyzed for this study are highlighted in bold.

After coding the interviews, a workshop with eight researchers from the Department of General Practice, who were not involved in the project, was held. In this workshop, we presented and discussed preliminary results with colleagues with different qualifications and professional backgrounds (e.g., sociologists, nursing, physicians). Using the theories of Berger/Luckmann (30) and Schütz/Luckmann (31) on social construction of reality and structures of the Life-World, the changes in the subjectively experienced reality and the associated social relationships (e.g., in the professional context or in socially significant vis-à-vis situations) reported by the interviewees were jointly analyzed. The theories mentioned analyze daily participation, which is particularly characterized by involvement in active communication processes. These meaningful processes changed in the respondents due to the experienced long COVID related limitations and lead to behavioral changes in the previously lived social contexts and to a changed reality vis-à-vis other people (31). Thus, these sociological theories provide an appropriate basis for the preparation and understanding of the interview data.

In the results chapter, the descriptively presented main statements of the interviewees are substantiated with original quotes from the interviews. Interviews were translated into English using forward translation. “P” at the end of the quotations stands for participant.

## Results

### Description of dataset

Overall, 27 people responded to the call for participation in this study. In the end, 25 interviews were conducted. One person withdrew his consent to participate without giving any reason and one person canceled the interview appointment due to health-related reasons. Participants were aged 21–67 years, with a median of 44 and a mean of 45 years. Of the 25 individuals, 18 were female (72%) and seven were male (18%). With the exception of two college students, one housewife, and one retiree, all participants were employed. Four of the employees and one student were on sick leave at the time of the interview. Overall, 10 participants reported that they had dropped out of work or their study programme for several months (*n* = 8), or permanently (*n* = 2), as a result of long COVID symptoms. Unlike many other countries, Germany has a social security system for employees including paid sick leave. An overview of sociodemographic information and selected occupational details of the participants is provided in Table 2 (sorted by interview date).

**Table 2:** Overview of sociodemographic information and occupational activities of the participants.

**Participant**	**Gender**	**Age**	**Occupation**	**Interview date (setting)**
P1	Female	30 years	Physician in residency training	01/24/2022 (online)
P2	Male	32 years	Management Consultant	02/17/2022 (online)
P3	Female	58 years	Housewife	02/18/2022 (online)
P4	Female	47 years	Clerk in a district adult education center	02/21/2022 (online)
P5	Female	44 years	Strategic Communication Officer	02/25/2022 (online)
P6	Female	25 years	College student	03/07/2022 (online)
T7	Male	45 years	Physical therapist at a geriatric early rehabilitation center	03/08/2022 (online)
P8	Male	49 years	Musicologist and social pedagogue	03/18/2022 (online)
P9	Female	37 years	Climate Protection Manager	03/24/2022 (online)
P10	Female	48 years	Secondary School Teacher	03/28/2022 (online)
P11	Male	67 years	Retiree	03/29/2022 (online)
P12	Female	56 years	Secretary	03/30/2022 (online)
P13	Male	21 years	College student	04/01/2022 (online)
P14	Female	52 years	Specialist nurse /pediatric intensive care	04/04/2022 (online)
P15	Female	60 years	Municipal official in nature conservation	04/05/2022 (online)
P16	Female	64 years	Physician Assistant	04/07/2022 (online)
P17	Male	37 years	Service technician in wind energy industry	04/12/2022 (online)
P18	Female	54 years	Prophylaxis specialist youth dental care	04/14/2022 (online)
P19	Female	25 years	Health care and nursing assistant	04/20/2022 (online)
P20	Female	55 years	Nurse	04/26/2022 (online)
P21	Female	23 years	Trainee speech therapy and therapy science	04/28/2022 (face-to-face)
P22	Female	48 years	Teacher	05/03/2022 (face-to-face)
P23	Male	63 years	Work preparation and project supervision in construction	05/03/2022 (face-to-face)
P24	Female	42 years	Administrator	05/11/2022 (face-to-face)
P25	Female	31 years	Physician internal medicine	05/14/2022 (online)

The interviews had a mean duration of 38 min (min: 17 min, max: 57 min). The content analyses comprised topics that were categorized in four inter-related categories: living situation, daily routine, occupational situation, and personal interests. Participants reported a wide variety of symptoms including fatigue, breathing problems, pain, and cognitive impairment that impacts their ability to pursue their lifes as they were used to before long COVID.

### Living situation and difficulties in daily activities

Participants reported that daily tasks such as self-care, childcare, household duties, or gardening represent a major challenge to them. Some interviewees described that they already have considerable difficulties with supposedly simple activities such as reading cooking recipes, preparing meals, maintaining body hygiene, or climbing stairs. “*Mostly I can go up one floor well, sometimes I can go up two, sometimes I can only go up half a floor*” *(P20)*. After performing such tasks, some participants described a pronounced fatigue symptomatology. For several respondents, these limitations led to a feeling of helplessness, dependence on people from their social environment, and regression in their personal development. In addition, patterns of attribution of these inabilities to one selves were evident in the statements. Several participants needed support in coping with daily activities and even in caring for themselves.

“*I need help in the household, too, I can't do it alone, it doesn't work” (P18)*.

“*It's also a little bit complicated. And that is that since the infection, I've lived with my parents again because I was unable to take care of myself in my apartment alone. […] Accordingly, I stayed all the time with my parents until a week ago now, I've tried to return to my own apartment several times already. But that does not work for me when I'm alone. I have also tried this in between, and I can't manage to take care of myself alone, I need help. And because there were always appointments to which I had to be driven, partly with a wheelchair or something, I always needed someone to help me. Currently, I'm in my apartment, but my parents are also here, as it were. I would actually like to look for household help, but that's not so easy either” (P25)*.

### Limitations in performing personal interests

Nineteen of the 25 participants stated that as a result of their health conditions, they experienced significant limitations in personal interests such as sports and cognitive activities, attendance at cultural and community events, and interacting with friends or meeting new people. Some interviewees were no longer able to engage in several of the leisure activities that were meaningful to them. These included physically demanding hobbies such as playing tennis, jogging, and dancing, but also less strenuous activities like going for walks, attending musical and theatrical performances, or singing and reading. In addition to long COVID symptoms, fear of re-infection and the public restriction to mitigate the COVID-19 pandemic were also described as reasons for these limitations.

“*You've asked about my leisure activities. Sports, I am a very athletic person. I was a track and field athlete, I did gymnastics, and I was also a dancer. Since then, none of this is possible any longer, that is, nothing at all. That means pursuing any sports is completely out of question. […] Other leisure activities? I liked to write texts, but I can't do that very well anymore either. […] I think young people suffer from it in a different way and more strongly, because I keep asking myself where does this lead to in the future? Ideally, I would like to combine all my interests and skills later” (P13)*.

This statement also illustrates that the experienced health and social limitations lead to a negative perception of the future scope of action and thus be a trigger for worries and fears. A further interviewee explained that, as a result of his COVID-19 condition, he had to give up his role of active sportsman and take on the role of passive spectator.

“*My leisure activities are sports, actually, different things. Bouldering, climbing, hiking, fitness classes, inline skating. None of that works anymore. That leaves me watching sports on TV when something like the Olympic Games or the biathlon or similar comes on. Apart from that, I still like to do a lot of sewing. But I can't do that anymore. I put that off until later, until sometime when I can do more again, hopefully. What else do I like to do? I also like to cook. I can't do that anymore either. I save that for later. Reading is only possible to a certain extent, sometimes. Mostly only in the evenings, and not like I used to” (P25)*.

As a result, many participants felt that they could no longer pursue some of the activities that had special value in their lives. In particular, younger participants in this study struggled with the loss of spontaneity and flexibility.

“*I lack the aspect of physical exercise and I don't see myself as being as flexible and spontaneous as I would like to be at 23, that I'm just like 'and now I'll be going out again tonight and it's not a problem if I don't sleep that many hours' or 'I feel like doing this or that sports activity now'. But actually, I know that if I do this now, the next 3 days are not really feasible. That's definitely why I can't do everything I want to do on that level” (P21)*.

While some other respondents list activities and personal interests that they can no longer pursue, this person explicitly emphasizes that she lacks the practice of a specific activity (physical exercise). This underlines the emotional connection to this activity, its importance for her quality of life, and her perception of this limitation as a great loss. In addition, a self-revelation regarding a changed self-image and body image is visible in this statement. This is also indicated in the following statement of another participant.

“*Before my illness, I was very active and had many obligations in addition to or just along with work, but also (during work). […] Now I lie down most of the day, and before, I was up on my feet all day” (P25)*.

Only one person stated that long COVID had not resulted in any changes in the pursuit of personal interests.

### Occupational situation

Work had a high priority in the lives of many of the participants. Most of the respondents were generally satisfied with their job situation prior to the illness and indicated that they had a supportive working environment. In addition to being unable to work for several months and resulting sick leave or entirely dropping out of working life, participants reported a reduced ability to cope with stress in daily working life and a reduction of workload or a change to an area with a lower workload.

“*[I was transferred to a position] at my work where the workload is much smaller than the normal daily work life on the ward. And that is stressful enough at the moment, so I still can't manage my normal work like that” (P1). “I notice that I am just not as resilient as I was before the corona infection and cannot be present as that might be necessary” (P14)*.

Several participants reported that, as a result of the inability to work or other occupational limitations, they had either no contact at all, only very limited contact, or no physical contact with colleagues, fellow students, or customers. “*Of course, some things have broken away […] and it has become more difficult to keep in contact with colleagues” (P2)*. In a few cases, the social environment became smaller or the number of face-to-face interactions decreased significantly. Other interviewees found it difficult to establish contacts with new colleagues or fellow students due to their limitations, which made them feel excluded and isolated. “*Socializing doesn't work out so well. […] I'm really all alone” (P13)*. These individuals reportedly feel they are missing out on opportunities and experiences that are important to their lives and careers. In addition, reduced social mobility affects their life and career planning as they no longer want to leave the familiar home and social environment or return to the environment in which they grew up. Several respondents perceive the inability to work due to long COVID as a great loss in their lives and point out how much they miss their jobs. “*I've never felt so much like going to work and fixing a windmill again. I actually kind of miss that” (P17)*. Some of the participants tried everything to return to work or to college and wished for nothing better than to be able to continue their work or student activities again. “*It was a daily struggle for me, so not a day went by where I didn't think about work” (P1). “That is my very greatest wish, to go back to my job” (P17)*.

For some participants, it was very difficult, or impossible, to envision an occupational perspective for themselves, which led to despair and pronounced stress situation. Those affected were no longer able to perform their current occupational activities. Simultaneously, due to the intensity and variety of their limitations, they also cannot imagine any other occupation that they can perform.

“*And I honestly don't have a plan B either, because in the current situation, I can't imagine tackling something else. To learn something new, to familiarize myself with another area. Let's say I can't physically work anymore, but I could sit in an office chair. If I were to sit in front of this computer, the training would already be [too much]. I can't think about it right now. I can't think of anything else right now except to get back on track first and then slowly increase, but I don't really see any perspective right now” (P17)*.

Every failed attempt to return to work was perceived as a sobering setback that intensified the despair and lack of perspective and thus unintentionally exacerbated the crisis. Long-term or permanent exclusion from working life as a result of health limitations can, as happened in the case of individual participants, set in motion a downward spiral that, without countermeasures, leads to a life crisis.

Some of the particpants completed a vocational reintegration program before returning to work. However, most of the individuals who returned to their jobs had to reduce their working hours, which led to a decrease in their income. “*This 20% that I'm now reducing, that's what's missing at my bank account. And no one compensates for that” (P12)*. Moreover, even under conditions of reduced working hours and reduced workload, performing an occupational activity rapidly brought respondents to their stress limits. This shows that the impact of long COVID on the occupational situation of those affected does not end when they return to work. “*First [I worked] only 2 h, then only 3 h. But I always realized if I spend half an hour, three quarters of an hour on the computer, oh God […] it did not work” (P18)*. Only two participants stated that long COVID had not affected their occupational situation.

### Support from the work environment

At the same time, eight interviewees stated that they perceived emotional support and understanding for their current condition by superiors and/or colleagues. Seven participants perceived the possibility of working from home as supportive which enabled them returning back to work, protect themselves from re-infection with COVID-19, and reduced the burden resulting from outside influences, such as distractions from co-workers. “*For me, going into the home office was a bit of self-protection […]. I just found it a relief. I could work […] and could also protect myself and others” (P4)*. Furthermore, participants reported that they could better structure their work in the home office, adjust it to their energy levels or doctor's appointments, and were more likely to be spared when additional tasks were assigned. “*I like to take advantage of the opportunities that I can then just switch off for appointments” (P2)*.

Contrary, five participants explicitly emphasized that they experience little consideration and support from their work environment. Respondents reported about colleagues or superiors who neglected or ignored their symptoms and made demands that they could hardly fulfill with their current state of condition. “*And then there are also people who forget, who then call me in again after all, and I have to say “it's inconvenient for me, but I can't do it anymore right now”” (P14)*. One person even experienced that colleagues complaining about the increased workload they had because of her illness. “*I also had two colleagues who complained about me to the staff council, along the lines of “I always have to cover for her because she is sick”” (P22)*. These employees worked in different sectors such as public administration, inpatient healthcare (hospital), or education (school). The lack of sensitivity from their work environment represented an additional burden, which further complicated their situation. Furthermore, individual interviewees reported that protection measures against COVID-19 infection such as masks and distance regulations were ignored by superiors and colleagues which caused a perception of lack of safety at work. One respondent stated that her supervisor questioned the health consequences of COVID-19 and doubted the existence of COVID-19 for ideological reasons, which prevented the recognition of her limitations.

“*In my case, the superior slightly favors the direction of negating everything and rejecting everything and then positions himself more on the side of those who are rebelling against the state and against this whole regulation” (P12)*.

### Role conflicts, identity crises, and “new reality with long COVID”

As a result of their symptoms, some of the interviewees were confronted with role conflicts and identity crises in different areas of life. These areas included partnership, relationship with children and parents, reputation among acquaintances and friends, occupational position, and self-image regarding body, mind, and social skills. Limitations were perceived in identity-forming attributes such as performance, self-determination, mobility, flexibility, mental and physical fitness, openness, or helpfulness. After reflecting on these limitations and comparing them with the situation before long COVID, some respondents identify significant changes regarding their own personality. “*I am actually someone who likes to be very social and help and support and be there for friends. I realize that I can't offer the help that I would otherwise like to offer when someone of my friends is ill or there is a birthday” (P1). “Before I had no restriction at all, I was always a very freedom-loving person, very self-determined” (P20). “Before my illness I was very active and did a lot […]. Now I lie down almost all day” (P25)*.

Due to these changes, roles such as the “power woman,” who works full-time and manages childcare, or the “main breadwinner” of one's family could no longer be fulfilled. This is shown in the quotation from a male participant who, due to his strong limitations in daily life, no longer met his self-image as a good partner and father. He questioned the importance of his role in the family and thus his social identity: “*I no longer feel like a main pillar in the family. I am the father, biologically in any case, but at the moment I feel pretty worthless […]. Of course, that's not true, I know that, but you just don't feel like a good partner and father anymore if you can't be the way you want to be and would like to do that with your family. You're just not the same anymore (P17)*.

Many of the interviewees were forced to adjust their lives, their daily routines, and also their own expectations to a new reality with long COVID. As a result, some of them felt that they were no longer the person they were before the disease and that they had lost a part of themselves. Partially, these changes were also confirmed to them by relatives. “*My husband wasn't there when I had my acute Corona infection, he was on a cure. And when he came home, he realized for the first time, ‘Okay, my wife, who was always such a doer,’ that it didn't work anymore. So, doing just didn't work and he had to be much more involved and that's still the case now. […] He is the one who has to do more in the partnership. And the things we have in common, the hobbies we share, the things we both enjoy, are limited to things where I don't have to physically exert myself” (P14)*. Similar to this person, other respondents also stated that the distribution of roles in the partnership changed and that their partners had to take over many or even all household or childcare tasks. “*In fact, because of the illness, it was now for months that my husband really did everything, had to do everything, because I couldn't. […] So going out with the children, taking children to kindergarten, picking up children, shopping, household chores, laundry, vacuuming, everything” (P1)*.

One woman reported that she decided against having children because of her low energy level. Thus, for her, long COVID affects her family planning, a decision that has a huge impact on the future shape of life and the perception of her identity and roles. “*We don't have children, I deliberately decided against having children because I realize that I'm busy enough with what we have. Children in addition to everything else is beyond me” (P9)*. For one person, the limitations resulted in a breakup of his partnership, the loss of friends, and a relocation to his father's home. In this case, the social environment changed, several relationships ended, and the interviewee, in middle age, again assumed the role of the son to be supported. “*I moved out […] at the request of my girlfriend. […] I moved in with my father for the time being. That is also an experience of moving back in with my father when I was still 45” (P7)*.

Furthermore, interviewees were also concerned that they no longer able to meet the expectations of others and themselves regarding their professional performance. One participant struggled with the fact that she could no longer meet her own demands for her work speed. “*I've always been a person with a quick grasp of things, and I've been trained to be efficient. […] I write a sentence and change my verbs three times in the sentence and then realize that the whole sentence order is no longer correct. That means I have to keep re-reading. I'm totally annoyed with myself. It's still like that” (P9)*. For another interviewee, the comments of some people about her reduced number of hours, aimed at a non-fulfillment of their benefit claims, posed an emotional burden. “*They always ask me ‘what? You're still not working much?’ […] Then the conversation is already over. I'm too sensitive for that” (P15)*. One person stated that some people in his environment could not understand why he so strongly wanted to return to his occupation and could not enjoy the benefits of his situation. “*I would really like to go back to work and others tell me to enjoy the time at home and just relax” (P17)*. In all three cases, the self-image of the respondents is fundamentally challenged by the limitations and the reaction of some people in their environment to it.

## Discussion

### Main results activities of daily life

A key finding of this qualitative study of 25 people with long COVID in Germany is that many interviewees face major challenges in pursuing activities of daily life such as climbing stairs, household chores, or caring for children, which they are unable to conduct without support. Furthermore, we found that ~3 out of 4 respondents experienced significant decline in personal interests. While some participants lost interest in certain leisure activities and adapted their needs to these new circumstances, others tried to maintain interests in which they experienced significant limitations or longed for those. These impairments in daily life and leisure activities were perceived as a considerable loss of quality of life.

### Comparison with other empirical studies and theoretical literature

Vuillemin et al. (32) showed that physical activity during leisure time has a positive effect on health-related quality of life. A substantial decrease in health-related and general quality of life in people with long COVID is also indicated by other studies (3, 7, 9, 17). Further research found that hospitalization due to COVID-19 results in reduced health-related quality of life and long-term psychological symptoms (e.g., depressive and anxiety symptoms) (33–35). According to Schütz (31), the daily living environment is based on a *familiar sequence of unproblematic experiences*. However, if the habitual sequence of these routines is questioned, the *chain of self-evidence* is interrupted (31), which can result in great uncertainty. This became evident in the interviews when respondents described that routine household activities, childcare, work, and leisure activities they are used to suddenly could no longer be completed without difficulties. The limitations caused by long COVID also have far-reaching consequences for the *recipe knowledge* of those affected, as described by Abels (36). Familiar certainties in the logic of the health care system, such as the doctor knows what measures to take for convalescence in patients, become fragile. The lack of disease-related knowledge that would allow comparison with the own disease course (e.g., the common knowledge that the common cold debilitates me temporarily before I am fully recovering and be healthy again) combined with lack of therapeutic approaches or own measures that eventually lead to a recovery provokes a feeling of powerlessness and helplessness. The new challenges resulting from long COVID create uncertainty in the assessment of the daily practical knowledge previously believed to be incontrovertible.

### Key findings occupational situation and comparison with further studies

For the 21 employed participants and the two students, the manifold limitations in their work or student life due to long COVID represented a strong burden. Eight of them have been out of work for several months, two of them even permanently. As a result, feelings of aimlessness, lack of perspective, insecurity, and uselessness became apparent. Similarly to our study, several participants from another sample also reported a change in their occupational status due to persistent health impairments after a COVID-19 infection (16). The umbrella review by Nittas et al. (9) cites various studies in which a substantial proportion of respondents with long COVID were unable to work for at least 8 weeks. At just under 50%, the proportion in our study is slightly smaller than in the study with the highest rate of work absence, which was reported to last several months (70%) and slightly above the level of some other surveys (3, 7). However, comparability is limited by different demographic sample structures and provision of healthcare and comprehensiveness of social system structures may vary among different countries. An analysis of German health insurance data from 2020 and 2021 shows that people diagnosed with a post-COVID-19 condition (persistent symptoms for at least 12 weeks) were reported unable to work for an average of more than 3 months in the year following their infection (37).

Some participants in our study were strongly committed to returning to work and perceived every failed work attempt as a sobering setback that increasingly led to despair and a lack of perspective. The fact that some respondents successfully returned to work while others did not, may have to do with differences in occupational activities and work environments, in addition to discrepancies in the severity and type of symptoms. Another key finding of this study is that the impact of long COVID on occupation does not end when people managed to return to work. Even when individual probands have successfully completed vocational reintegration with reduced working hours, they experience ongoing struggles with reduced performance and fatigue. This shows that the burden long COVID remains after returning to work and affected individuals continue to feel dysfunctional. This finding has been also described in other publications (6, 38). This highlights that reintegrative measures should not end once people with long COVID returned to work. Employers and care providers should acknowledge needs and facilitate access to work-related interventions, such as fatigue and energy management training [e.g., pacing (39, 40)], counseling and occupational therapy, for those affected and support them in adapting the work environment and content to their needs and resources (40). Our study indicated, that people with long COVID benefited from flexible working time models that they can tailor to their needs and prevent them from exhaustion. Additionally, awareness of coworkers and superiors were experienced as a positive resource. Raising awareness and providing information to employers might contribute to better working experiences for people with long COVID conditions.

In this research, decreased incomes were reported due to decreased working hours that had to be adjusted to the reduced energy levels. Some participants in other studies reported also changes in their working tasks (3, 7). Rajan et al. (41) point to significant economic consequences for people with long COVID, their families, and the overall society. In this study, decreased incomes were only described by a few people, and problems such as the threat of dismissal or the risk of poverty were not an issue. This may be related to the social security system in Germany, which includes elements such as paid sick leave, sickness benefits, and a reduced earning capacity pension in the case of occupational disability.

Several interviewees from our sample stated that, as a result of the inability to work or other occupational limitations, the social environment became smaller or the number of face-to-face interactions decreased. Social consequences such as isolation or stigmatization due to a long absence from work are also reported by other studies which suggested to facilitate access to state vocational and retirement counseling and to provide support through social services or rehabilitation institutions (6, 7, 16).

### Comparison with theoretical literature

Berger and Luckmann (30) provided an explanation for the high emotional strain resulting from the limited ability to work. According to them, everything related to work is the most important *zone* of a person's daily world. Participation in this *zone*, which is of such significance to an individual, is determined by what that person has done, is currently doing, can do, and will do in the future in this *zone* (30). Many people with long COVID in our study were unable to make an impact in their working lifes, due to their experienced symptoms. This resulted in an experience of reduced or even complete lack of self-efficacy.

### Main results support from the work environment

Many respondents stated that their superiors and work colleagues supported them regarding their condition. At the same time, several participants emphasized that they experience little support in their work environment. For example, colleagues or superiors made demands to them knowingly that they could not fulfill or overwhelm them. The affected interviewees worked in different sectors such as public administration, healthcare, or education, which indicates that this phenomenon is not limited to certain professions. The additional experienced lack of sensitivity exacerbated the already precarious and highly stressful situation for the respective participants. Health insurance data from Germany indicate that there are significant differences between some occupational groups in terms of the risks for sick leave with a post COVID diagnosis. Metal workers but also people employed in social and educational, and the health sector had considerable higher odds for sick leave than those employed in administrative or social science occupations (37).

### Relief through home office?

A total of seven respondents in this research perceived the possibility of working from home as supportive which enabled them to return to work, protect themselves from re-infection with COVID-19, and reduce the burdens. A facilitation through home office is primarily found among participants with screen-based workplaces in their main occupation. For some of the respondents, especially those working in the healthcare sector, home office was by the nature of this occupation not a viable option. Working from home was also more difficult if young children or school-age adolescents have to be cared for at home during the pandemic (42). However, similar to some other studies on home office during the COVID-19 pandemic with people with and without chronic illnesses (43, 44), most employees from this study who were offered this opportunity perceived working from home as helpful. Thus, if operationally possible, employers may consider providing people with long COVID the ability to work from home. At the same time, employees should pay attention to allow interacting and networking with colleagues as this has been shown to be an important resource.

### Summary social aspects and role conflicts associated with long COVID

A key finding of this study is that with long COVID, “social symptoms” occur in addition to disease-related symptoms. These include limitation or loss of social functioning, role conflicts, and identity crises in the context of family, friends, and occupation, changed self-image in terms of body and mind, and reduced horicontal social mobility. The interviews revealed that for many participants, restrictions in their social life are predominant and continuous compared to biomedical symptoms, and social interaction is most severely restricted. This reduces the scope of action and social reach of affected persons. As a result of the ongoing impairment of daily life and family interaction, many individuals become aware that they are no longer as they were before they had COVID-19, which can lead to role conflicts (3, 11, 45). Such role conflicts can result in an identity crisis, for example, due to a lack of experience of self-efficacy as a result of a prolonged period of incapacity for occupation. Long COVID is associated with a variety of social burdens such as insecurity and role pressure and, in addition to the current life and occupational situation, it also affects life and career planning. In contrast to many other (chronic) medical conditions, long COVID people are left uncertain about what will happen next and what disease-related and social limitations will affect them in the mid- and long-term. These uncertainties of those affected are exacerbated by inadequate treatment concepts and long waiting times for rehabilitation services. The lack of prospects for a successful recovery creates fears about the future regarding occupational activity or education, and social stress within the family environment. These described uncertainties, role conflicts, and social burdens should be given increased consideration in the treatment and management of long COVID.

The study adds to the scientific literature a description of “social symptoms” of long COVID and the impact of the disease on social functioning, perception of own identity, and roles in the context of partnership, family, and work environment of affected individuals. For decision-makers in national policies and healthcare, the study shows a need for action regarding the development of tailored concepts for outpatient care and support of people with long COVID in their home environment (support in coping with the disease in daily life, self-care, household care, childcare) and permanent occupational reintegration [development of a specific reintegration program, adaptation of workplaces, provision of relief, and support services in daily work life (e.g., work-related therapeutic measures)].

### Strengths and limitations

The results we presented refer to a sample of 25 adults whose recruitment occurred in a limited period of time. Although we did not reach saturation, we are convinced that we could identify and characterize main aspects and challenges that long COVID patients in Germany are experiencing in their daily and work life. Regarding the sample size, we followed existing literature on good qualitative research practice, which recommends conducting at least 12 interviews (21–23). Our sample consisted mainly of participants from one region in Germany. Furthermore, most respondents could be characterized as working middle-class people of German descendent. As we offered both video teleconference and face-to-face interviews, we may have reached participants that otherwise would not have participated e.g., due to limited mobility or lack of video teleconference equipment. Various population groups (e.g., people with a migration history, older adults, people with a low educational level, or people with a speech disorder) may get disadvantaged to participate and are underrepresented in this study. Further research should take these underrepresented populations into account. Due to the applied exclusion criteria were participants were to be excluded if they were not able to pursue a 1-h interview, this study may have neglected people with very high burden of symptoms. Moreover, participants reported retrospectively on perceived changes in their lives and occupations. The researchers had no information about the actual changes in the participants' life situation or occupational status, therefore bias in reporting and disclosure of the actual status by participants cannot be ruled out. A strength of this study is that within the framework of qualitative interviews with open questions, a greater depth in content could be generated and the life reality of participants can be approached more closely than it would be possible with solely quantitative surveys.

## Conclusions

Besides biomedical symptoms, people with long COVID face severe social limitations that strongly impair them in pursuing daily activities, personal interests, and occupational life. Role conflicts and identity crises in different areas such as daily life, occupational status, or self-image regarding body and mind may result. Especially the temporary or permanent inability to work, including failed attempts to return to work, and the continuing limitations after vocational reintegration causes uncertainty and despair on the side of the participants. To prevent people with long COVID from isolation, job loss and resulting crisis, decision-makers should consider strategies to systematically support people with long COVID in their working environment and foster reintegration into the workforce. Elements of such a strategy may include:

1. Measures addressed to employers and managers to raise awareness regarding possible key challenges of an occupational activity with long COVID,
2. Improved access to relief services such as demand-oriented vocational reintegration or work-related therapeutic interventions,
3. A target-group-specific expansion of the right to home office,
4. Financial support for employers to create long COVID-sensitive workplaces,
5. Government assistance for people who, due to their health impairments, are affected by decreased incomes following a reduction in working hours or a change of activity.

Furthermore, access to support services for daily routines such as household help or childcare should also be improved. These points represent only initial approaches to the search for measures to address the problems identified in the interviews with people with long COVID in their personal environments and in their occupational context. In the development of a long COVID strategy, further issues must be considered. Overall, society, research, politics, the healthcare system, employers, and fellow citizens are called upon to pay more attention to the topic of long COVID and to take the situation of people with the disease seriously. In particular, long COVID should be viewed more as a “social disease.”

## Data availability statement

The raw data supporting the conclusions of this article will be made available by the authors, without undue reservation.

## Ethics statement

Written informed consent was obtained from the individual(s) for the publication of any potentially identifiable images or data included in this article.

## Author contributions

TS prepared the first draft. SR conducted the interviews. TS, JK, AD, GK, and SR participated in the analysis of the data. FM, JK, AD, GW, GK, and SR revised the manuscript and provided further contributions and suggestions. All authors read and approved the final manuscript.
