# Supplementary material for: How do long COVID patients perceive their current life situation and occupational perspective? Results of a qualitative interview study in Germany
Source: Front Public Health. 2023 Mar 9;11:1155193. doi: 10.3389/fpubh.2023.1155193 (PMC10034079; doi:10.3389/fpubh.2023.1155193)
Supplement: Supplementary file 1 [file Data_Sheet_1.pdf]

Interview guideline long COVID

Version III (February 18th, 2022)

Demographic data

Gender:

Nationality:

Age:

Occupation:

Marital status:

Children yes/no; Number (if "yes"):

A Health situation

I. Do you remember when you first became aware that you had an ongoing limitation after your corona infection? What were your thoughts and feelings regarding this?
II. What health limitations do you currently have?
III. Do you have the impression that people around you are considerate of your current health limitations and if so, what do you attribute this to?
IV. Have you received a corona vaccination despite your infection with the coronavirus?

B Private / social environment and everyday experiences

I. Describe your situation at home. What is your daily life like in your family/partnership?
II. How do your health limitations currently affect your interactions with family, friends and your partner?
III. Are there people in need of care in your environment whom you take care of?
IV. Are you active in the church and what significance does it have for you in your everyday life?
V. What are your hobbies and how do you manage to implement them in your daily life?

VI. Do you do sports regularly?

VII. During the corona pandemic, there were government restrictions such as contact restrictions, distance regulations, and masking requirements. How did you experience these stipulations?

VIII. In the corona pandemic, some people strictly followed the corona rules, while others showed more of a negative attitude and questioned the extent of the corona crisis. What are your thoughts on this? Has your opinion or behavior changed after you infected yourself with the coronavirus?

C Occupational context

Please tell me about your current occupation and the collegial environment in which you work.

I. Has your occupational situation changed due to health limitations following the corona infection? If so, what is different or new?

II. How did you experience the period of the corona pandemic in your occupational environment?

III. Do you have the possibility to implement home office and how did you manage this to the corona pandemic?

IV. How did you experience your supervisors in dealing with the corona pandemic?

V. How do you manage to combine your current health limitation with job requirements?

D Perception of healthcare in Germany / corona pandemic

I. How do you experience the medical support or advice regarding your current health limitation?

II. Do you know other people who have been infected with the coronavirus and have similar symptoms as you? Do you have any contact with these people?

III. How do you currently feel about the federal and state governments in terms of dealing with the corona pandemic?

IV. What has been your experience regarding education on vaccination for corona protection?

E Life perspective

I. The corona crisis has been going on for over two years now. What has changed in your circle of friends during this time? What has remained the same?
II. Do you feel that you can currently do the things that are important and meaningful to you?
III. What strategy have you found for yourself to deal with your current health limitation?
IV. Looking back at the time before you were infected with corona virus, would you behave differently looking back at your health limitations today?
